# Fabrication of 3D Controlled *in vitro* Microenvironments

**DOI:** 10.1016/j.mex.2014.06.003

**Published:** 2014-07-08

**Authors:** Berrin Ozdil, Sevgi Onal, Tugce Oruc, Devrim Pesen Okvur

**Affiliations:** Izmir Institute of Technology, Department of Molecular Biology and Genetics, 35430 Izmir, Turkey

**Keywords:** UV lithography, Polydimethylsiloxane, Microfluidic device, Lab-on-a-chip, Microenvironment, Hydrogel, Matrigel

## Abstract

Microfluidics-based lab-on-a-chips have many advantages, one of which is to provide physiologically relevant settings for cell biology experiments. Thus there is an ever increasing interest in their fabrication. Our goal is to construct three dimensional (3D) Controlled *in vitro* Microenvironments (C*iv*Ms) that mimic the *in vivo* microenvironments. Here, we present our optimized fabrication method that works for various lab-on-a-chip designs with a wide range of dimensions. The most crucial points are:

• While using one type of SU-8 photoresist (SU-2075), fine tuning of ramp, dwell time, spin speed, durations of soft bake, UV exposure and development allows fabrication of SU-8 masters with various heights from 40 to 600 μm.
• Molding PDMS (polydimethylsiloxane) at room temperature for at least two days instead of baking at higher temperatures prevents not only tears and bubbles in PDMS stamps but also cracks in the SU-8 master.
• 3D nature of the C*iv*Ms is ensured by keeping the devices inverted during gel polymerization.

## Graphical abstract

**Figure:**
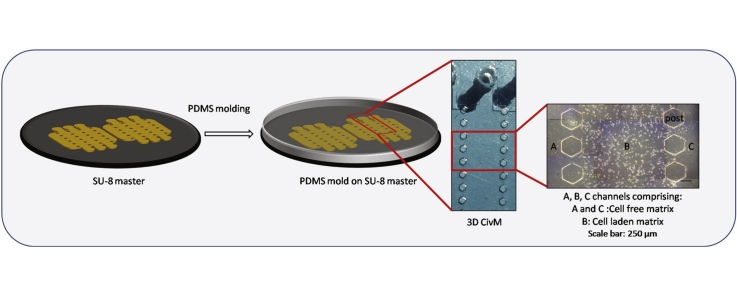

Microfluidics-based lab-on-a-chips have many advantages [1]: Small volumes down to pL are used. Small volumes provide enhanced safety when dangerous or toxic chemicals or biological agents are used. Precise spatial and temporal control can be achieved. High throughput analysis is facilitated [2]. Fabrication costs are low. The devices are portable. Finally, the devices provide physiologically relevant settings for cell biology experiments [3–8]. Such advantages have resulted in an increased interest in the methodological details of fabrication of lab-on-a-chips [9–11].

## Method details

### UV lithography

UV lithography (UVL) which is also called photolithography is a parallel writing method for fabrication of 2D and 3D micrometer scale designs using photo-reactive materials, called photoresists [10]. There are two types of photoresists: Positive and negative. Positive photoresist is degraded by exposure to UV light followed by dissolution in a developer while negative photoresist such as SU-8, is cross-linked in the same process. SU-8 is widely used for fabrication of masters that are in turn used for both 2D and 3D structures of interest. SU-8 is an epoxy based negative photoresist. SU-8 is available in different viscosities and is categorized as SU-8 2000 and 3000 series. The higher the viscosity (and the number following ‘SU-8’), the higher the thickness of the polymer spun on a surface. We fabricate SU-8 masters with heights between 40 and 600 μm using SU-8 2075. These masters can then be used for PDMS molding. PDMS molds in turn are used for fabricating 3D Controlled *in vitro* Microenvironments (C*iv*Ms). Some of our 3D microfluidic platforms have a set of microfluidic channels separated by an array of posts. Such systems are convenient for studying different hydrogels and cell types in the same device at predefined dimensions while mimicking *in vivo* conditions [2–5].

UV lithography is carried out in a Class 1000 clean room. Special lab overalls suited for clean room use are worn.

First improvement of our method is the ability to generate SU-8 layers with different thicknesses ranging from 40 to 600 microns using only SU-8 2075 through careful optimization of the steps of UV lithography, in particular the spinning step. Thus the users do not need to procure all different kinds of SU-8 in their laboratories.  

**Materials**

Photoresist SU-8 2075 [!**Caution**: Wear protective gloves].
SU-8 developer (Stored at +4 °C)
Si wafer
Acetone
Isopropanol
Dust-free tissue paper
Aluminum foil
Paper towel
Designed mask
Tweezers

**Equipment**

Hot plate
Mask aligner
Spin coater [!**Caution**: Do not open lid until the spinner comes to a full stop]
Fume hood
Stereoscopic microscope

#### Spin coating of SU-8

##### Day 1

First set the hot plate to 65 °C at least half an hour beforehand to ensure uniform heating and place the SU-8 bottle on the bench so that its temperature equilibrates to room temperature.

*A piece of aluminum foil should be placed on the hot plate before placing the wafer to avoid any photoresist residues contaminating the hot plate and to facilitate handling of the wafer. In addition, the tweezers used for handling SU-8 should not be used for handling other materials.*

*-* Take a silicon wafer using tweezers from its package and leave it on the hot plate for approximately 5 min, then pick up the wafer with its aluminum foil and place it on the bench.
- Pour the SU-8 onto the wafer holding the SU-8 bottle very close to the wafer surface to prevent the formation of bubbles.

*Slowly retract the SU-8 bottle by rotating it and place again in the hood but do not close its mouth with its cap. Loosely cover the mouth of the bottle with a piece of aluminum foil and wait until all the SU-8 moves back towards the bottom of the bottle. Any SU-8 remaining on the mouth of the bottle will crystallize in time and can interfere with a uniform SU-8 coating on the silicon wafer.*

- Disperse the SU-8 on the wafer homogenously by gently moving the wafer at an angle in a circular motion. Avoid generating any bubbles or waves.
*-* Keep the wafer on the bench for approximately 10 min so that it equilibrates to room temperature and the photoresist relaxes.

*Relaxation of the photoresist can alternatively be carried out on the chuck of the spin coater. This ensures smaller temperature differences between the chuck and the sample and a homogenous surface during various spin rates.*

- Cover the inner surface of the spin coater with aluminum foil beforehand to keep the spin coater clean.
- Use the proper recipe that will yield the desired thickness of the SU-8 layer.

*For instance: For a final SU-8 thickness of 200 μm, perform the following steps:*

*Ramp up to 500 rpm in 5 s, spin at 500 rpm for 5 s*,
*ramp up to 1000 rpm in 5 s, spin at 1000 rpm for 20 s*,
*ramp down to 500 rpm in 5 s, spin 500 rpm for 5 s*,
*ramp down to 0 rpm in 5 s.*

- Wait until the spinner comes to a full stop before opening the lid.
- Remove the wafer from the spin coater and place it on a piece of aluminum foil on the bench to allow for the relaxation of photoresist. Any waves present will slowly disappear.
- Place the wafer with its aluminum foil on the hot plate set to 65 °C for 20 min. Then increase the temperature to 95 °C and leave the wafer at this temperature for 5 h. This is the soft bake step. If thin SU-8 layers are prepared, 3–4 h are enough.
- Dispose of the materials contaminated with SU-8 according to your institution's guidelines.

#### Exposure of the SU-8 coated wafer to UV light

##### Day 2

First set the hot plate to 95 °C at least half an hour beforehand to ensure uniform heating.

- To test whether any wrinkles will form and to confirm that the soft bake is complete, place the SU-8 coated wafer on the hot plate at 95 °C. If there are no wrinkles on the SU-8 surface, then the sample is ready for UV exposure. If wrinkles appear, place the SU-8 coated wafer on the bench for the relaxation of the photoresist for approximately 5 min and then re-place it on the hot plate for an additional bake of 10 min. Repeat these steps until no wrinkles form.
- Based on the power settings of the mask aligner, one can calculate the time for exposure for a desired final dose (mWatt/cm^2^ s = mJ/cm^2^). For a setting of 8 mWatt/cm^2^, we used exposure times up to 60 s.
- Adjust the time of the exposure to 60 s for an SU-8 thickness of about 400 μm, and to 30 s for thicknesses less than 200 μm. Here, SU-8 is intentionally overexposed to facilitate PDMS removal in later steps. However, too much overexposure will prevent the proper development of the SU-8 pattern.
- Place the SU-8 coated wafer on the mask aligner stage. Then place the acetate film mask on the wafer. The opaque surface of the mask should face the SU-8 layer.
- After UV exposure is completed, place the sample on the bench for 5 min for relaxation of the photoresist.
- Place the sample on the hot plate set at 65 °C for 5 min, then increase the temperature to 95 °C and leave the wafer at this temperature for about 15 min. This is the post bake step. Turn off the hot plate and leave the sample on the hot plate to let it cool down slowly to room temperature.

#### Development of the SU-8 master

##### Day 3

- Place the SU-8 developer and isopropanol on the bench so that they equilibrate to room temperature.
- Keep the SU-8 master in a petri dish filled with developer for 5 min without shaking. Then shake the sample in the developer for 15 min. After this, dispose of the developer. Shake SU-8 master in a fresh volume of developer again for 20 min. The UV exposed parts of SU-8 will remain on the wafer and the unexposed parts will be washed away.

*If the pattern has posts (pillars) on a thin SU-8 layer, treat the sample with SU-8 developer for 10-15 min, i.e. shorter durations, and check that all the pillars are developed well under a stereo microscope with a UV filter. Even if only one pillar region is not open (developed), this may cause absence of a PDMS post in turn and thus leakage of the hydrogels through the adjacent channels during the CivMs experiments.*

- Apply the isopropanol (IP) test. When a few drops of IP are applied on a small part of the SU-8 sample, usually the corner of a pattern, a white precipitate will form if the SU-8 is under-developed. If this is the case, shake the sample again in a fresh volume of developer. If the sample is well-developed, *i.e.* there is no white precipitate, hold the sample vertically and wash it 10 times with developer to remove any remaining small SU-8 particles on the wafer, and then wash it 10 times with IP which stops the development.
- Dry the SU-8 master with dust-free tissue paper. The SU-8 master is now ready.
*-* Wash the petri dishes and tweezers with acetone, IP and finally with H_2_O.

*Remember that SU-8 is sensitive to light. All the applications on the wafer with SU-8 should be performed in a clean room which is illuminated with yellow light. After the SU-8 master is ready, it can be handled in a standard laboratory.*

### PDMS molding

Second improvement of our method is for PDMS molding through room temperature polymerization, which not only preserves the SU-8 masters for years but also prevents damage to the resulting PDMS molds.  

**Materials**

Sylgard 184 silicone elastomer base and curing agent
Demolding agent: Triton-X-100:H_2_O:Absolute EtOH 1:9:40
Plastic cups and spoons
Aluminum foil and paper towel

**Equipment**

Balance
Vacuum desiccator

PDMS is provided as base and curing agent. The typical ratio for mixing is 10:1. A 5:1 ratio results in a stiffer PDMS.

- Determine the final weight of PDMS needed and calculate the required weight for base and curing agent. Weigh the base first and then add the appropriate amount of curing agent which is easier to weight. For a four inch wafer, a total of 30 g of PDMS is sufficient.
- Mix the base and curing agent well.

*The high number of bubbles reflects how good the base is mixed with the curing agent*.

- Degas the mixture to remove all the bubbles by placing the mixture in a desiccator coupled to vacuum for 2× 10 min.
- In the meantime, wash the SU-8 master with EtOH (70%), and H_2_O. Then clean it with the demolding agent (cleaning buffer). Demolding agent provides easy separation of PDMS mold from the SU-8 master in later steps.
- Use a 10 cm glass petri dish to shape a piece of aluminum foil into a shallow container. Place a piece of double sticky tape in the middle and place the SU-8 master inside.
- Pour the degassed PDMS mixture onto the SU-8 master.
*-* Leave the PDMS mixture on a uniformly level surface for polymerization at room temperature for at least 2 days.

*If the PDMS mixture on the SU-8 master is baked just after it is poured on the wafer, any possible bubbles generated during the pouring of the PDMS mixture will be fixed in the PDMS and the SU-8 master will be more likely to crack.*

- After at least 2 days, separate the polymerized PDMS from the wafer.

*Applying EtOH at the PDMS – SU-8 interface helps removal*.

### Construction of 3D Controlled *in vitro* Microenvironments (C*iv*Ms)

Construction of 3D Controlled *in vitro* Microenvironments needs to be preceded with the fabrication of SU-8 masters and molding of PDMS. Bonding of glass slides and PDMS molds is required for the completion of the 3D devices. Fabricated devices should be well sterilized to prevent any contamination that may hinder the biological application. SU-8 masters are reusable while the devices themselves can also be cleaned and reused although this is neither required nor recommended.

Third improvement of our method is that keeping the devices inverted during gel polymerization ensures a truly 3D distribution of cells in the matrix. Otherwise cells sink the bottom glass surface and show a 2D phenotype. In addition, we provide a detailed procedure for a rather neglected step of cleaning of the PDMS molds as well cleaned PDMS molds are essential for proper formation of 3D microenvironments that are devoid of any contaminants.  

**Materials**

Glass slides
Scotch tape
70% EtOH
Deionized water (H_2_O)
Matrigel

**Equipment**

Sonicator
UV/Ozone Plasma Cleaner [!**Caution**: Do not inhale the gases generated during the process].
Hot plate

#### Preparation of PDMS molds

- Cut out the PDMS molds along their borders and punch holes at proper positions for inlets and outlets.
- Use Scotch tape to remove any dust from the PDMS surfaces.
- Holding the PDMS molds with plastic tweezers, wash them with H_2_O several times and place them into glass containers such as beakers.
- Sonicate in H_2_O for 10 min; rinse with H_2_O 5 times.
- Sonicate in 70% EtOH for 5 min; rinse with 70% EtOH twice.
- Keep in 70% EtOH for 5 min on bench.
- Place the samples inside a laminar hood, rinse with H_2_O once and aspirate any liquid left on or inside the samples.
- After the PDMS molds are dry, place them into an autoclaved petri dish; the patterned sides of the PDMS molds should be facing up. Cover the petri dish with aluminum foil.
- Keep these samples at room temperature for 2 days so that they are completely dry as the next step is bonding and the samples that will be treated in UV/ozone plasma should be completely dry.

#### Permanent bonding of 3D C*iv*Ms

*-* Treat a clean slide and a PDMS mold in the UV/ozone cleaner for 5 min. Then immediately bond the treated surfaces facing each other to obtain the complete 3D C*iv*Ms.

*At each UV/ozone treatment, clean one slide and one PDMS mold as the bonding step should be done immediately without losing the effect of the UV/ozone treatment.*

- Place the 3D C*iv*Ms on the hot plate at nearly 100 °C for at least 10 min and cover them with elevated aluminum foil pieces to create an oven effect, to protect from dust and to ensure permanent bonding of the PDMS molds with the slides.
- Turn off the hot plate and let the 3D C*iv*Ms cool down to room temperature.

#### Sterilization of 3D C*iv*Ms

- Rinse all inside and outside surfaces of the 3D C*iv*Ms and the petri dish with 70% EtOH and take them into a laminar flow hood.
- Aspirate any liquid on or inside the 3D C*iv*Ms and wash inside the channels with autoclaved H_2_O twice.
- Aspirate any liquid on or inside the 3D C*iv*Ms and place them into a new autoclaved petri dish.
- Let the samples dry and expose them to UV light for 30 min.
- Place the 3D C*iv*Ms inside the petri dish covered with aluminum foil in an oven and heat the samples at 80 °C for 24 h for restoration of hydrophobicity.

*During UV/ozone treatment, the PDMS and glass slide surfaces become hydrophilic. In order to make them hydrophobic again, and thus, prevent the leakage of the hydrogels through the adjacent microchannels during loading, the samples are heated at 80 °C for at least 24 h (4). Once this heating process is completed, the samples are ready for loading of the hydrogels.*

#### Loading of 3D C*iv*Ms with hydrogels

*-* Mix Matrigel with cell suspension at 1:1 ratio on ice.

*A rack made of aluminum placed on ice is very useful for holding tubes at a constant and cold temperature of +4 °C*.

*Matrigel is normally stored at −80 °C. Thaw the matrigel overnight within ice bath at +4 °C. Other hydrogels such as collagen can also be used instead of matrigel*.

*-* Place the 3D C*iv*Ms directly on 70% EtOH soaked sterile filter paper placed on an aluminum block in contact with an ice bath.

*If the 3D CivMs are not cold, matrigel will start to polymerize upon contact and loading can be compromised*.

*-* Load the cell laden Matrigel to the corresponding channel with a 200 μl-pipette and allow for polymerization at room temperature for 30 min. Invert the samples to prevent cells from sinking to the bottom glass surface.

*While loading the gels, hold the sample vertically and work slowly to prevent the gel of interest from passing through pillar regions to other channels. Inverting the 3D CivMs just after loading a (cell-laden) matrix makes the borders of gels more defined and ensures that cells do not precipitate to the bottom of the device.*

- After gel loading and polymerization are complete, add culture media into the medium reservoirs.
- Place the 3D C*iv*Ms into new sterile petri dishes and place open microcentrifuge tubes filled with autoclaved H_2_O to minimize the evaporation of medium from the devices. Also close inlets and outlets of the gel channels with PDMS pieces to minimize evaporation.
- Keep the samples at 37 °C and 5% CO_2_ or other cell culture conditions required by the cells.
- Collect data on cell behavior, for example, by taking phase contrast or fluorescence images of cells in 3D C*iv*Ms every day. Once image data are collected, Photoshop and/or ImageJ can be used for image processing and analysis.
